# Study protocol for a target trial emulation: CHoosing the right diAlysis Modality in clinical PractIce: hemOdiafiltratioN or hemodialysis (CHAMPION)

**DOI:** 10.1186/s12882-026-05315-z

**Published:** 2026-08-28

**Authors:** Sanne Roos, John Larkin, Linda H. Ficociello, Len A. Usvyat, Yue Jiao, Luca Neri, Vivi Zhou, Menno Brandjes, Peter J. Blankestijn, Michiel L. Bots, Saskia Haitjema, Marianne C. Verhaar, Roemer J. Janse, Robin W. M. Vernooij

**Affiliations:** 1 Department of Nephrology and Hypertension, University Medical Center Utrecht, Utrecht University, Heidelberglaan 100 Utrecht, 3584 CX The Netherlands; 2 Central Diagnostic Laboratory, University Medical Center Utrecht, Utrecht University, Utrecht, The Netherlands; 3 Renal Research Institute, New York, NY USA; 4 Ortec Logiqcare B.V., Zoetermeer, The Netherlands; 5 Julius Center for Health Sciences and Primary Care, University Medical Center Utrecht, Utrecht University, Utrecht, The Netherlands

**Keywords:** Hemodiafiltration, Hemodialysis, Target trial emulation, Mortality, Hospitalizations

## Abstract

**Background:**

Randomized studies have demonstrated that high-volume hemodiafiltration results in reduced mortality compared to conventional hemodialysis treatment. However, eligibility criteria in these trials may limit generalizability to routine clinical practice. Some of these trials reported a limited number of events, underscoring the need to further evaluate the effect of hemodiafiltration on mortality. We will conduct a target trial emulation study using data from routine clinical practice. The primary aim of this study is to evaluate whether high-volume hemodiafiltration reduces all-cause mortality. The secondary aim is to assess cause-specific mortality. Other aims include assessing all-cause and cause-specific hospitalizations, as well as cumulative length of hospital stay and the dose–response relationship between convection volume in hemodiafiltration and the outcomes.

**Methods:**

Data will be obtained from the second version of ApolloDialDb (Apollo), an anonymized dialysis dataset capturing over 1000 variables from patients from all over the world. For this study, we will include adult patients from European countries with kidney failure who initiated with at least one treatment of high-flux hemodialysis or hemodiafiltration between 01 January 2018 and 30 June 2024, and who were prescribed a thrice-weekly dialysis schedule at the start. Patients starting with home dialysis will be excluded. We will use a target trial emulation approach with a clone-censor-weight design and marginal structural models, controlling for selection bias, survivor bias, and competing risk bias. Sub-analyses will be performed to investigate the effect of high-volume hemodiafiltration (≥ 23 L of convection volume). Inverse probability weighting will be applied to adjust for predefined confounders including sociodemographic, clinical, and anthropometric factors, as well as comorbidities to achieve balance between treatment groups.

**Discussion:**

In addition to randomized studies, prior large observational studies have indicated a survival benefit for hemodiafiltration, as well as a possible reduction of hospitalizations. The target trial emulation study outlined in this protocol will expand this knowledge and provide generalizable insights on the effects of hemodiafiltration on outcomes by using real-world data representative of routine clinical practice while appropriately addressing sources of bias.

**Graphical abstract:**

**Figure:**
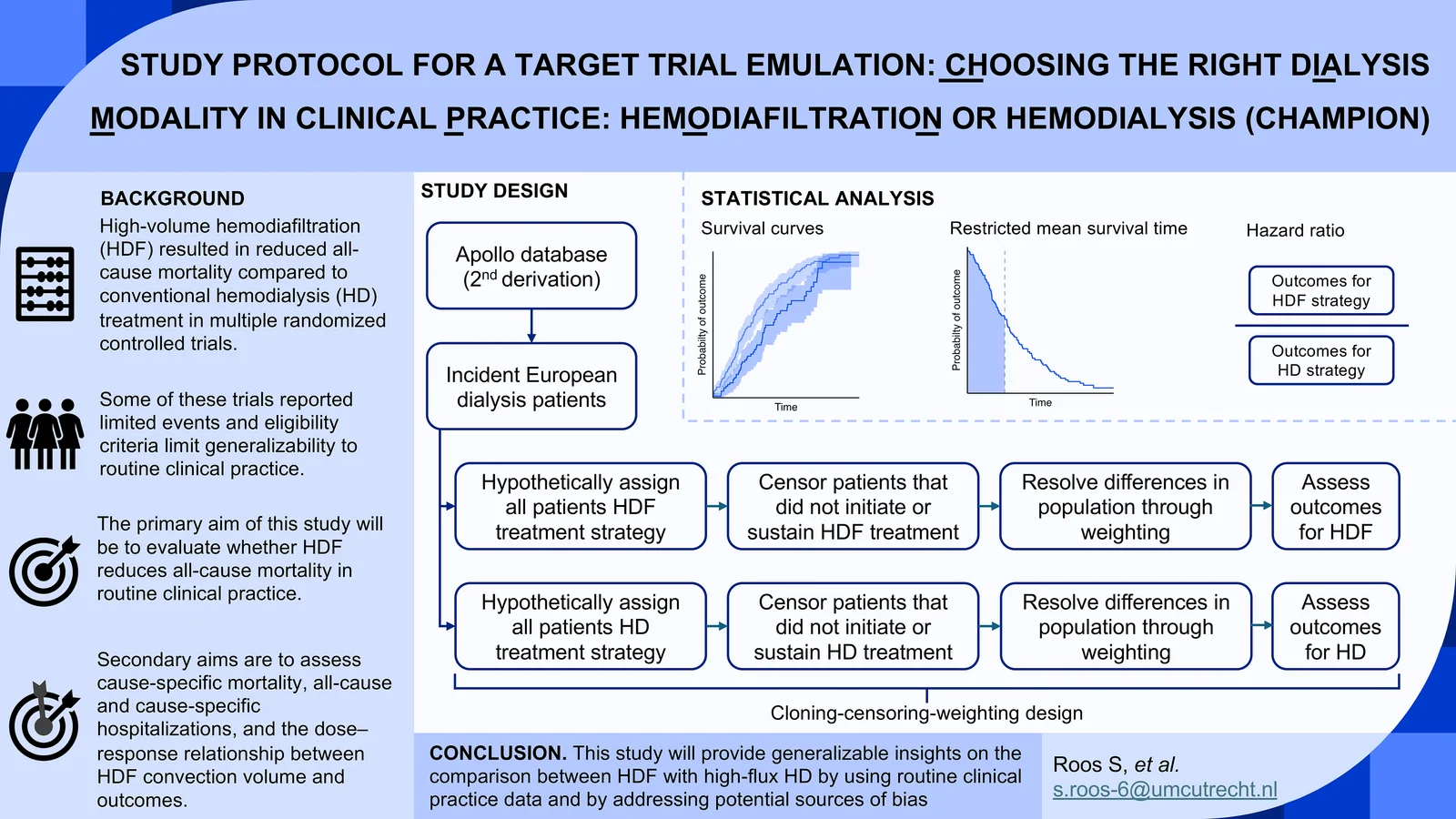

**Supplementary Information:**

The online version contains supplementary material available at https://doi.org/10.1186/s12882-026-05315-z.

## Lay summary

This protocol outlines a study in which we will examine the effects of hemodiafiltration (HDF) compared with high flux hemodialysis (HD) using data from standard day-to-day dialysis care, collected from across Europe. Clinical trials have previously shown that HDF provides benefits for survival and quality of life. However, it remains uncertain whether these benefits apply to all patients or only in healthier patients, who meet the eligibility criteria to participate in a clinical trial. We will use advanced statistical methods, specifically target trial emulation, to closely mimic a randomized clinical trial using real-world data and thereby reduce bias. The study will evaluate overall 5-year survival, causes of death, hospitalizations, and the impact of higher HDF convection volumes to help guide future dialysis care decisions.

**Supplementary Information:**

The online version contains supplementary material available at https://doi.org/10.1186/s12882-026-05315-z.

## Background

The CONVINCE randomized controlled trial (RCT) demonstrated that high-volume hemodiafiltration (HDF) improved survival compared with standard high-flux hemodialysis (HD) [1]. More recently, an individual patient data meta-analysis that combined CONVINCE with four previous randomized trials showed a further reduction in mortality risk with increasing convection volumes [2]. These effects were consistent across patient and treatment characteristics.

However, eligibility criteria in these trials may limit generalizability to routine clinical practice. In CONVINCE, patients were enrolled only if a convection volume of at least 23 L per session was considered achievable. The other trials applied several strict inclusion and exclusion criteria, which excluded people with an active infection or systemic disease, malnutrition, uncontrolled anemia, or those taking any immunosuppressive therapy [3–5]. Consequently, the findings may not fully represent outcomes in the broader dialysis population. Several observational studies have also indicated a survival benefit and a reduction of hospitalizations with HDF [6–11]. However, observational studies are often confounded by several forms of bias (immortal time or survival bias, prevalent user or selection bias) because of their design.

Target trial emulation addresses this gap by applying a RCT framework to observational data, thereby enabling causal inference within observational data captured in and representative of routine clinical practice [12]. The primary aim of this predefined study protocol is to evaluate whether high-volume HDF reduces all-cause mortality compared to HD in routine clinical practice, and the secondary aim is to assess cause-specific mortality. Other aims include assessing all-cause and cause-specific hospitalizations, as well as cumulative length of hospital stay and the dose–response relationship between HDF convection volume and outcomes.

## Methods

### Datasource

Data will be obtained from the second version of ApolloDialDb (Apollo), an anonymized global dialysis dataset across 41 countries. This database contains longitudinal data on dialysis treatments from a global kidney provider network (Fresenius Medical Care, Bad Homburg, DE) [13, 14]. This second version of Apollo contains data from 113,415 patients on dialysis from Europe [14]. This includes demographic and laboratory data, dialysis specific data as well as medication use, and clinical diagnoses. Data from dialysis sessions or mortality occurring in the hospital during a hospitalization throughout the dialysis course has been registered from the respective hospital and is included in the database.

### Ethics

A rigorous anonymization process was applied to ensure compliance with privacy regulations (e.g., General Data Protection Regulation in the United States, Health Insurance Portability and Accountability Act in Europe and other applicable local laws) and to minimize the risk of patient reidentification. The anonymization strategy was developed following a reidentification risk assessment by Privacy Analytics an IQVIA company (Ontario, Canada) and was based on established privacy standards and expert methodologies [13]. 

### Study design and patient selection

This study will emulate a predefined clinical trial using the target trial emulation framework. In this framework, a RCT is simulated by systematically analyzing observational data using design and principles of RCT as template [12]. This framework includes eligibility criteria, treatment strategies, treatment assignment, study initiation and follow-up, outcomes, causal effect estimands and statistical analysis [15]. The protocol for this target trial is detailed in Table 1. A comparison with the details from the protocol from CONVINCE trial can be found at Supplemental Table 1.

**Table 1:** Detailed study protocol for target trial emulation

Protocol component	Description of target trial	Description of emulation
	**Eligibility criteria**	
Inclusion criteria	1. Patients of ≥ 18 years old, with kidney failure 2. Initiate with high-flux HD or HDF in a European country where both HD and HDF can be offered. 3. Initiate within a time frame where at least 6 months of follow-up would theoretically be possible, with a maximum of five years 4. At baseline, there is the intention to follow a thrice-weekly schedule	1. Patients of ≥ 18 years old, with kidney failure 2. Initiate with high-flux HD or HDF between 1 January 2018 and 30 June 2024 in a European country where both modalities are represented in the available data 3. Patients prescribed a thrice-weekly schedule at the start
Exclusion criteria	1. Initiating with hemodialysis or hemodiafiltration at home	1. Initiating with hemodialysis or hemodiafiltration at home. 2. No data available within 14 days after dialysis initiation*
	**Treatment strategies**	
	1. Initiate HDF within the first 90 days after dialysis initiation (grace period) and continue HDF throughout the grace period receiving at least three HDF treatments every two weeks. 2. Initiate HD within the first 90 days after dialysis initiation (grace period) and continue HD throughout the grace period receiving more than three HD treatments every two weeks.	Same as target trial
	**Assignment procedures**	
	• Randomization between HDF and high-flux HD	• Randomization is emulated by cloning each individual and then assigning each clone to one of the treatment strategies
	**Follow-up**	
	• Starts at the randomization (assignment of a strategy, just before dialysis initiation) • Ends at death, informative censoring due to a competing event (kidney transplantation, recovery of kidney function, withdrawal from dialysis), administrative censoring (end of study or after 5 years) or loss-to-follow-up, whichever came first.	• Starts at the emulated randomization (assignment of a strategy, just before dialysis initiation, or within 14 days after initiation*) • Ends at death, informative censoring due to a competing event (kidney transplantation, recovery of kidney function, withdrawal from dialysis), administrative censoring (31 December 2024 or after maximum follow-up^♠^) or loss-to-follow-up, whichever came first.
	**Outcomes**	
Primary outcome	All-cause mortality	Same as target trial
Secondary outcome	• cause-specific mortality	Same as target trial • cause specific events are identified from relevant ICD-10 codes
Other outcomes	• all-cause hospitalizations • cause-specific hospitalizations • Cumulative length of hospital stay • dose–response relationship between convection volume and all outcomes above, in patients receiving HDF	Same as target trial • cause specific events are identified from relevant ICD-10 codes
	**Estimands**	
	• Intention-to-treat effect • 5-year survival using a cumulative incidence curve • Restricted mean survival time • Hazard ratio (HR) for the outcome, presented per year for a total of five years	Per protocol-effect • 5-year♠ survival using a cumulative incidence curve • Restricted mean survival time • Hazard ratio (HR) for the outcome, presented per year for a total of five years
	**Analysis plan**	
	• Intention-to-treat analysis, see Follow-up for censoring.	• Per protocol effect, see Follow-up for censoring. • Analysis is conducted in expanded data set that includes two clones for each eligible patient (one for each treatment strategy). IPCW is performed, both for informative and artificial censoring within the grace period, and for informative censoring after the grace period.

*Although this may theoretically lead to some level of immortal time bias, excluding everyone who does not have any data at day one of dialysis initiation will lead to selection bias (due to missingness). As the database for this study contains treatment related data in an outpatient setting, hospitalization at the start could be the reason for missing data in the first period

♠ To protect patient privacy, only the calendar quarter of dialysis initiation is available. We will therefore assign the midpoint of the quarter as the initiation date, as this approach minimizes misclassification. As a result, the maximum follow-up period will be 4 years and 9 months

HDF, hemodiafiltration; HD, hemodialysis; ICD-10, International Classification of Diseases; IPCW, inverse probability of censoring weighting

Inclusion criteria for this study will be:

- Patients with age ≥ 18 years old at baseline;
- Patients with kidney failure who initiate dialysis and receive at least one high-flux HD or HDF treatment between 1 January 2018 and 30 June of 2024;
- Treatment occurs in a European country where both HDF and HD are available and where patients in the dataset receive both modalities each calendar year, ensuring representation of both treatments within each country;
- Patients prescribed thrice-weekly dialysis at the start.

Exclusion criteria will be:

- Patients starting with dialysis at home;
- Patients who do not have the first treatment-related data available within 14 days after dialysis initiation.*.

*Because this database captures outpatient treatment only, data at treatment initiation may be missing for patients who were at another dialysis provider or who were hospitalized at that time. Excluding these patients could therefore introduce selection bias. Including patients with available data within the first 14 days may introduce a degree of immortal time bias; however, we believe for 14 days this will be limited. We will perform subgroup analyses of patients who have first treatment data at dialysis initiation compared to patients of whom first data is available within 14 days but not at the first initiation.

### Treatment strategies

We will compare the initiation of HDF with the initiation of high-flux HD. Aligning study eligibility, treatment assignment, and the start of follow-up is essential to prevent immortal time bias. However, this alignment is challenging because many patients switch between HD and HDF over time, and patients often initiate incenter dialysis with HD and subsequently transition to HDF at a later stage, calling for an approach that can accommodate practice patterns and dynamic treatment strategies.

Therefore, we will implement two different methods of analysis. First, we will use the clone-censor weight (CCW) approach. This approach begins with the duplication each individual (creation of “clones”) at the start of follow-up (first dialysis initiation) and each clone will be assigned to either the HDF strategy or the HD strategy.

**HDF strategy:**

1. Initiate HDF within the first 90 days after dialysis initiation (grace period).
2. Continue HDF throughout the grace period (receiving at least three HDF treatments every two weeks).

**HD strategy:**

1. Initiate HD within the first 90 days after dialysis initiation (grace period).
2. Continue HD throughout the grace period (receiving more than three HD treatments every two weeks).

The 90-day grace period was selected based on the expectation that patients who transition to HDF generally do so within the first three months after dialysis initiation. Similarly, the definition of treatment continuation as receiving at least three HDF treatments every two weeks was based on clinical practice patterns. After assignment, clones are followed and censored in case they deviate from the assigned treatment strategy during the grace period of 90 days. In addition to this approach, we will use a Marginal Structural Model (MSM) to estimate the effect of sustained treatment with HDF versus sustained treatment with HD. For this model, follow-up will start at dialysis initiation, and treatment will be included as a time-varying covariate throughout the entire follow-up period.

Lastly, we will conduct additional analyses for both the CCW approach and MSM in which we will evaluate consistent treatment strategies, but with HDF redefined as achievement of high-volume HDF (≥ 23 L of convection volume). For the CCW approach, we will calculate the mean delivered convection volume for every two-week period within the grace period. For MSM, we will calculate the mean delivered convection volume for every six months.

### Interpretation and estimands

Both the CCW approach and MSM aim to estimate the average treatment effect (ATE) to quantify the causal effect of treatment strategies in the target population. However, the CCW approach compares the effect of initiating HDF within 90 days with not initiating HDF within 90 days, whereas the MSM compares the effect of sustained treatment with HDF versus sustained treatment with HD over the entire study period. In the CCW framework, treatment adherence is assessed during the predefined grace period, requiring full adherence to the assigned strategy within this window, irrespective of treatment changes thereafter.

### Outcomes

The primary outcome will be the cumulative incidence of all-cause mortality. Secondary outcomes will be cardiovascular mortality, infection-related mortality including COVID-19 and infection-related mortality excluding COVID-19. Additionally, all-cause hospitalization and cause-specific hospitalization, as well as the length of hospitalization (proportion of the follow-up time in days) will be analyzed. Standard International Classification of Diseases (ICD-10) codes will be used to determine these outcomes. ICD-10 codes that are used to determine the outcomes are summarized in Supplemental Table S2. Lastly, the dose-relationship between convection volume and outcomes will be investigated for patients receiving HDF, with convection volume analyzed as a continuous variable to preserve all information that would otherwise be lost through categorization.

### Statistical analysis

#### Sample size

No formal sample size calculation was performed, as all patients meeting the inclusion and exclusion criteria will be included in the study. We expect this sample size to be sufficiently large to answer the research questions, given the size of the Apollo database.

#### CCW approach

**Fig. 1:**
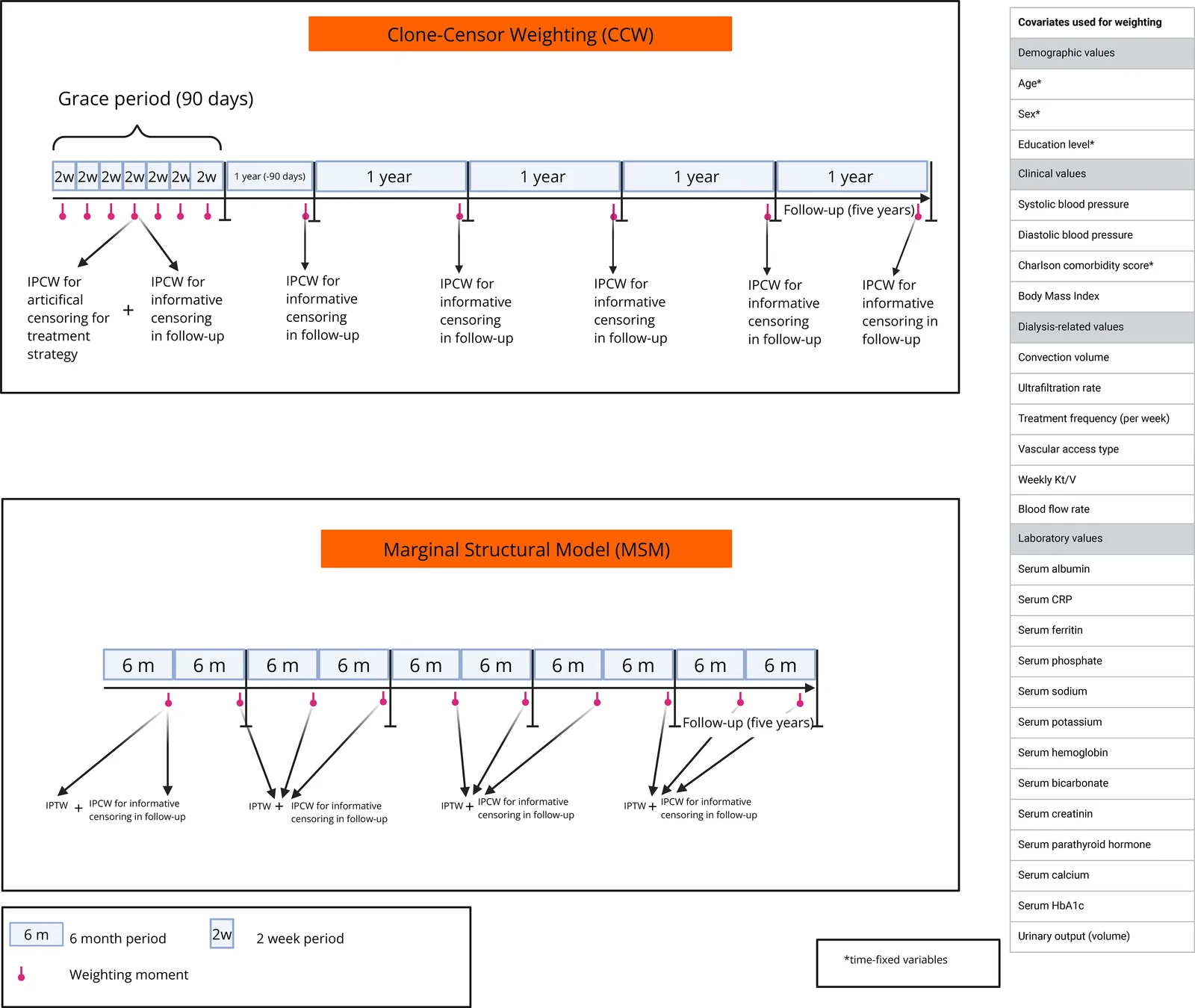
Weighting approach for Clone-Censor Weighting (CCW) and Marginal Structural Model (MSM). For the Clone-Censor-Weighting approach, weighting will take place every two weeks in the grace period of 90 days. Separate weights for artificial censoring for the treatment strategy, and for the informative censoring are calculated. Afterwards, weighting is repeated at the end of each year during the total follow-up of 5 years. For the Marginal Structural Model approach, weighting will take place every 6 months for the total follow-up of 5 years. Separate weights for treatment modality switches (IPTW) and for informative censoring are calculated. IPTW, inverse probability of treatment weighting; IPCW, inverse probability of censor weighting; Kt/V, metric for dialysis adequacy based on urea clearance (K), treatment time (t) and distribution volume (V); HbA1c, hemoglobin A1c

First, a stacked dataset will be created in which each individual is duplicated, creating one clone for each of the two treatment strategies. As a result, both treatment groups will be perfectly balanced at baseline. However, selection bias may arise after baseline because clones are followed over time and artificially censored when their observed data deviates from the assigned treatment strategy. Of course, patients can be censored both within the grace period and after the grace period due to competing risks, including kidney transplantation, recovery of kidney function or dialysis withdrawal. Both forms of censoring are likely to be informative. To address this issue, we will apply inverse-probability-of-censoring weighting (IPCW) using a generalized linear model (GLM) that includes covariates predictive of censoring and the outcome. We will determine weights for the grace period and the period thereafter separately. Weighting within the grace period will take place every two weeks, whereas weighting for the period after that will occur with yearly intervals. The probability of remaining uncensored will be the outcome of this model. To assess whether the weights adequately reduce covariate imbalance between the two arms due to artificial censoring during the grace period, we will calculate the standardized mean difference (SMD). A SMD above 10% will be considered evidence of residual imbalance, potentially indicating misspecification of the weight model [16]. The weighting approaches with the included covariates in each model of both CCW and MSM are summarized in Fig. 1.

**Fig. 2:**
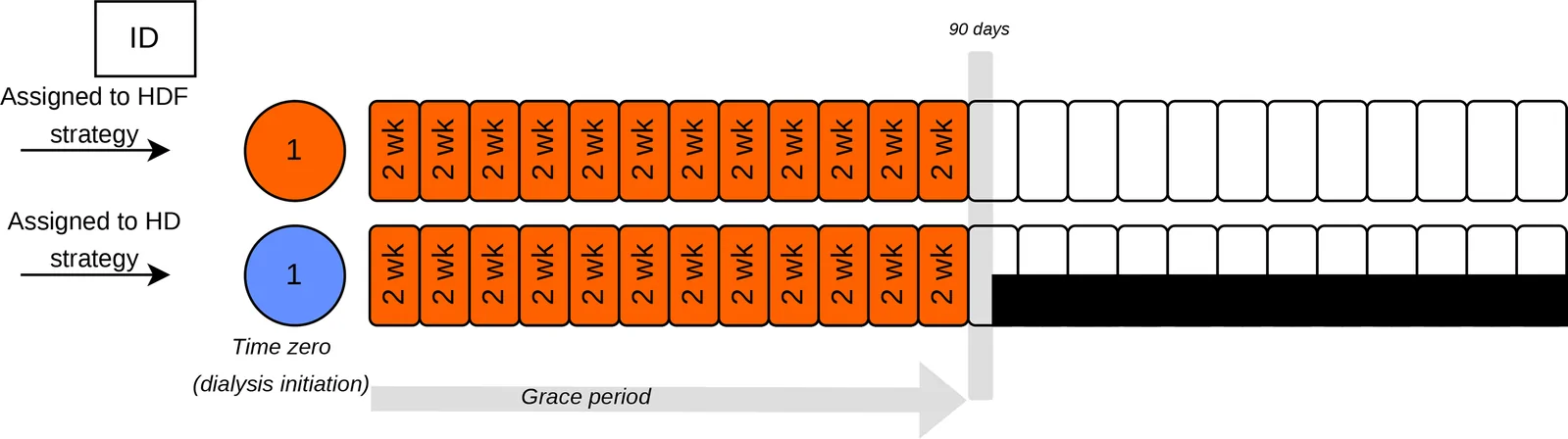
Examples of censoring for treatment strategy of clone censor weighting approach. **Example ID 1** The clone assigned to HD will be censored at 90 days, as this individual has not initiated HD within the grace period

At two-weekly intervals, we will determine whether clones are adherent to their assigned treatment strategies. Of note, an event only contributes to the arm in which the patient is still uncensored at the time of event. For the HDF initiation strategy, patients will be censored at 90 days if they have not initiated HDF, and vice versa for the HD strategy. Individuals who die within the grace period while having received only one modality will have their replicated observations contribute follow-up time and a death event to both treatment strategies. This approach reflects the assumption that these early deaths could have occurred under either treatment strategy, as no clear modality choice had yet been established. By attributing these early events to both arms, this design prevents immortal time bias. Examples of censoring for the treatment strategies are shown in Fig. 2.

**Example ID 2:**
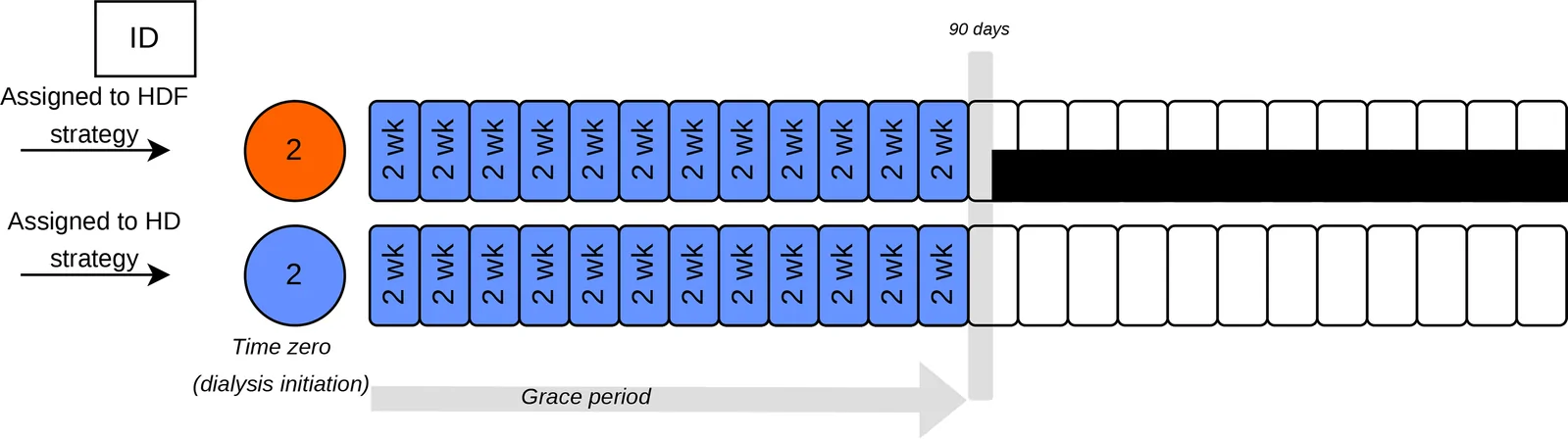
The clone assigned to HDF will be censored at 90 days, as this individual has not initiated HDF within the grace period

**Example ID 3:**
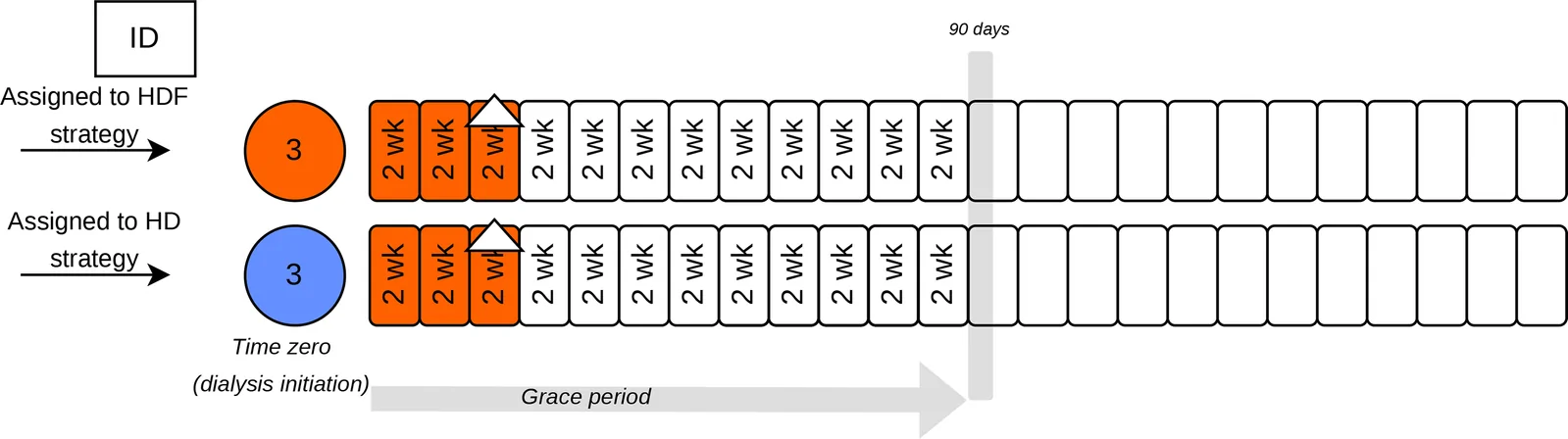
This death occurs within the grace period before a clear treatment choice could have been made, therefore this early death will count in both treatment strategies, to prevent immortal time bias

**Example ID 4:**
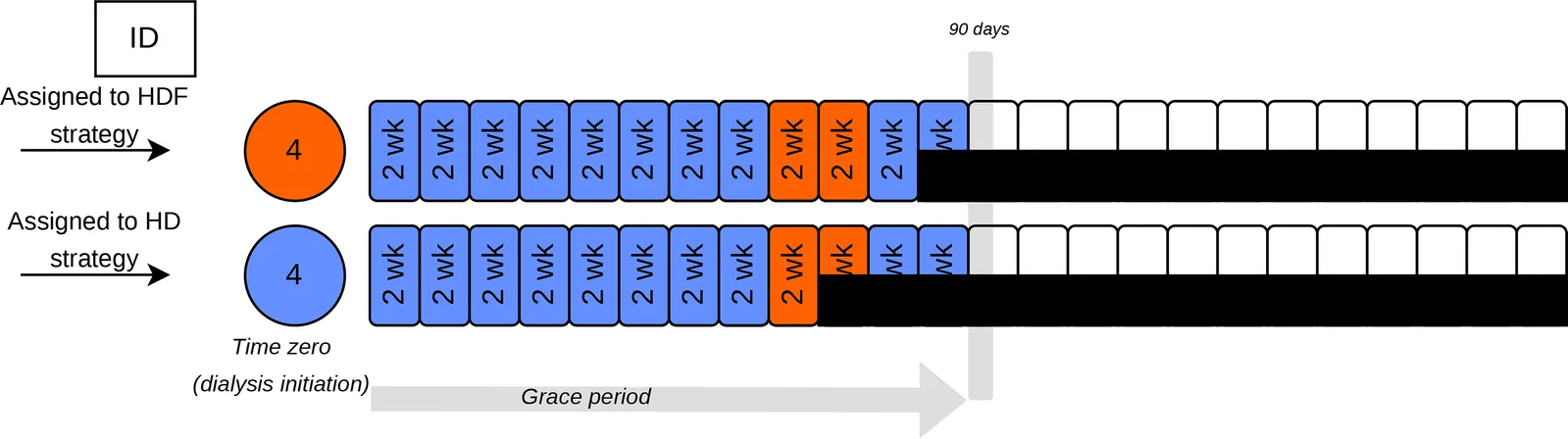
The clone assigned to HDF will be censored because the individual has started HDF, but not continued HDF for three times within each consecutive two week period within the grace period. The clone assigned to HD will be censored because the individual has started HD, but not continued HD more than three times within each consective two week period within the grace period

**Figure:**
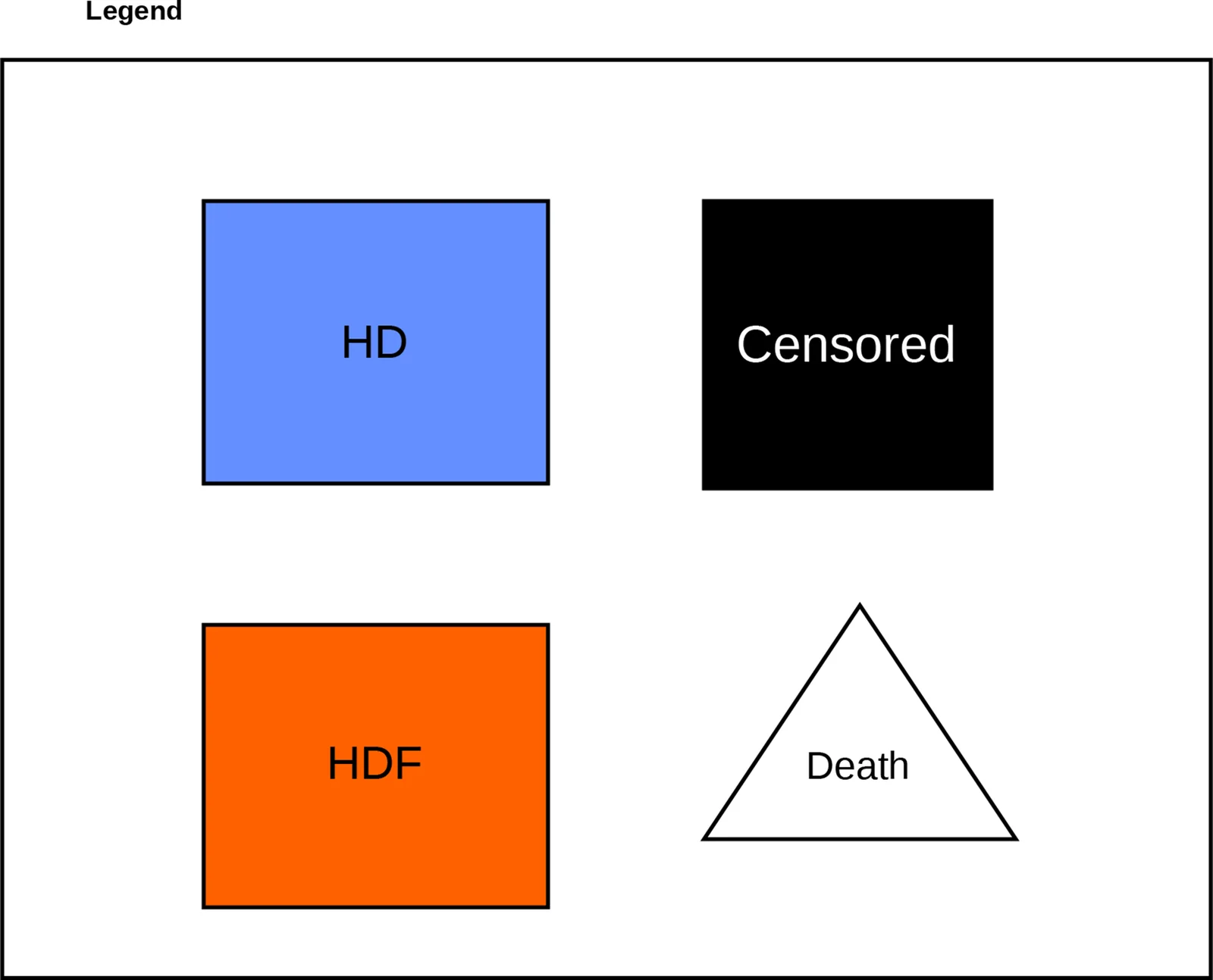
Each two weeks in the first 90 days, adherence for the assigned treatment strategy is assessed. Data from all individuals are duplicates so there are two clones. Each clone is assigned to either the HDF strategy (orange) or the HD strategy (purple). ID, patient identifier; HDF, hemodiafiltration; HD, hemodialysis; wk, weeks

The outcome model will be a weighted pooled logistic regression model fitted over all time intervals on the stacked dataset to estimate the effect of the two treatment strategies. Country will be included as a random effect to account for clustering at the country level, reflecting differences in treatment practices and the varying likelihood of receiving treatment across countries. To account for the within-individual correlation introduced by cloning, 95% confidence intervals will be estimated using nonparametric bootstrap resampling at the individual level with 500 samples, ensuring that both clones of each resampled individual are retained within each bootstrap sample. Both weighted cumulative incidence curves, restricted mean survival time, as well as hazard ratios for each year during a total of five years will be used to illustrate the differential effect of the two treatment strategies on outcomes.

#### Marginal structural model

To minimize confounding by indication, we will account for the possibility that patients with a more favorable clinical profile are more likely to receive HDF. Because treatment allocation and patient characteristics may change over time, time-varying confounding is expected. To address this, we will use inverse-probability-of-treatment weighting (IPTW). Treatment weights will be estimated at 6-month intervals using the probability of receiving the observed treatment and of having the outcome, conditional on measured covariates and treatment history. In addition, IPCW will be calculated at the same 6-month intervals to account for informative censoring during follow-up. The final analysis weights will be obtained by combining the treatment and censoring weights.

The distribution of the weights will be carefully evaluated to assess the presence of extreme values or excessive influence from individual observations. If excessive variability is observed, stabilized weights based on the marginal probability of treatment and censoring will be applied. If stabilization using marginal probabilities does not sufficiently reduce weight variability, alternative stabilization based on fixed-effects models will be used. In that case, the final Cox proportional hazards model will additionally be adjusted for baseline (time-fixed) confounders to account for factors incorporated in the fixed-effects stabilization procedure.

#### Missingness

We will impute missing data using longitudinal multiple imputation by chained equations (MICE), generating ten imputed datasets with 50 iterations each. Results will then be pooled using Rubin’s rules [17]. Imputation of all the relevant confounding covariates will be performed on the full study population before data duplication. Because the HDF and HD clones differ only with respect to censoring time and censoring reason, the dataset will subsequently be duplicated, after which censoring time and censoring reason will be assigned separately for each clone.

All analyses will be conducted using R statistical software (version 4.5.2).

## Discussion

The target trial emulation study outlined in this protocol will expand current knowledge regarding the effectiveness of HDF compared with conventional high-flux HD and provide generalizable insights on outcomes by using data representative of routine clinical practice and at the same time addressing sources of bias. By using a large international database representative of real-world clinical practice, this study aims to complement randomized trial evidence such as from the CONVINCE RCT and evaluate treatment effectiveness in a more heterogeneous, incident dialysis population.

An important strength of this study is the inclusion of incident dialysis patients. Previous observational studies evaluating HDF have often included prevalent dialysis populations, which are susceptible to prevalent user or survivor bias, as patients must survive long enough to receive the exposure of interest [7–11]. By aligning eligibility criteria, treatment assignment, and start of follow-up at dialysis initiation, this study design reduces bias. Furthermore, the use of both clone-censor-weighting and marginal structural modeling allows evaluation of different clinically relevant treatment strategies while accounting for time-varying treatment patterns and confounding.

Additional strengths include the large sample size and the granularity of the Apollo database, which contains detailed information on demographics, laboratory measurements, dialysis treatment characteristics and clinical diagnoses from every observation captured in thrice weekly dialysis care. These data will enable extensive adjustment for potential confounders.

Several methodological considerations should also be acknowledged. First, some protocol definitions and cut-off points were based primarily on clinical reasoning rather than strong empirical evidence. Although these choices may seem somewhat arbitrary, transparency regarding these design decisions is an important principle of target trial emulation and allows reproducibility and clear interpretation.

Second, the Apollo database only captures outpatient dialysis treatments from a single dialysis provider. Although it includes a large number of patients from different European countries, the focus on Europe and the use of data from a single provider may limit generalizability to other regions and healthcare settings. Furthermore, treatment-related data at dialysis initiation are unavailable for patients hospitalized during the first period. To reduce the potential for selection bias, patients with available treatment data within the first 14 days after dialysis initiation will be included. This approach may introduce a limited degree of immortal time bias, however, we considered this risk to be outweighed by the selection bias that could arise from systematically excluding patients requiring hospitalization shortly after dialysis initiation. We will perform subgroup analyses to evaluate the results in this group who does not have available data at the first treatment.

Finally, despite extensive adjustment strategies and the use of advanced causal inference methods, residual confounding cannot be fully eliminated, as is inherent to observational research. Some variables were not registered systematically such as ambulatory blood pressure measurements, functional status or cardiac function as measured on echocardiogram. Therefore, we could not include these into the analyses. Nevertheless, by explicitly emulating a target trial and applying causal analytic methods to a large real-world dataset, this study seeks to provide clinically relevant and more generalizable evidence regarding the effectiveness of HDF.

## Supplementary Information

Below is the link to the electronic supplementary material.


Supplementary Material 1


## Data Availability

All analysis code will be available at https://github.com/SRoos2025 All questions regarded the database may be directed to John.Larkin@freseniusmedicalcare.com.

## Abbreviations

HDF: Hemodiafiltration
HD: Hemodialysis
RCT: Randomized Controlled Trial
CCW: Clone Censor Weighting
MSM: Marginal Structural Model
ATE: Average Treatment Effect
ICD: International Classification of Diseases
IPCW: Inverse probability of censoring weighting
GLM: Generalized Linear Model
SMD: Standardized Mean Difference
IPTW: Inverse probability of treatment weighting
MICE: Multivariate Imputation by Chained Equations
