# Supplementary material for: Study protocol for a target trial emulation: CHoosing the right diAlysis Modality in clinical PractIce: hemOdiafiltratioN or hemodialysis (CHAMPION)
Source: BMC Nephrol. 2026 Aug 28;27:508. doi: 10.1186/s12882-026-05315-z (PMC13525684; doi:10.1186/s12882-026-05315-z)
Supplement: Supplementary file 1 [file 12882_2026_5315_MOESM1_ESM.pdf]

Supplemental files

Study protocol for a target trial emulation: CHoosing the right diAlysis Modality in clinical PractIce: hemOdiafiltratioN or hemodialysis (CHAMPION)

Sanne Roos, John Larkin, Linda H Ficociello, Len A Usvyat, Yue Jiao, Luca Neri, Vivi Zhou, Menno Brandjes, Peter J Blankestijn, Michiel L Bots, Saskia Haitjema, Marianne C Verhaar, Roemer J. Janse*, Robin WM Vernooij*

Table of contents

Supplemental Table S1: CONVINCE trial compared to target trial 3

Supplemental Table S2: ICD-10 codes for secondary outcomes... 5

Supplemental Table S1: CONVINCE trial compared to target trial

Protocol component	CONVINCE	Description of target trial
	**Eligibility criteria**	
Inclusion criteria	1. Patients of ≥18 years old with kidney failure<br>2. Treated with HD for at least 3 months<br>3. Deemed candidates for high-volume HDF (≥23 L in post-dilution mode per session)<br>4. Willing to undergo dialysis three times a week<br>5. Understanding of trial procedures and ability to adhere to trial protocol	1. Patients of ≥ 18 years old, with kidney failure<br>2. Initiate with high-flux HD or HDF in a European country where both HD and HDF can be offered.<br>3. Initiate within a time frame where at least 6 months of follow-up would theoretically be possible, with a maximum of five years<br>4. At baseline, there is the intention to follow a thrice-weekly schedule
Exclusion criteria	1. Severe non-adherence to the dialysis procedure and accompanying prescriptions, especially the frequency and duration of dialysis treatment<br>2. Life expectancy below 3 months<br>3. Previous hemodiafiltration treatment less than 90 days before screening<br>4. Anticipated kidney transplantation within 6 months after screening<br>5. Evidence of any other disease or medical condition that may intervere with the planned treatment<br>6. Unavailability for trial visits for 3 months or more	1. Initiating with hemodialysis or hemodiafiltration at home
	**Treatment strategies**	
	1. Start HDF with high convection volume (≥23 L per session)<br>2. Start high-flux HD	1. Initiate HDF within the first 90 days after dialysis initiation (grace period) and continue HDF throughout the grace period receiving at least three HDF treatments every two weeks.

		2. Initiate HD within the first 90 days after dialysis initiation (grace period) and continue HD throughout the grace period receiving more than three HD treatments every two weeks.
	**Assignment procedures**	
	Randomization between high volume (≥ 23 L per session) HDF and high-flux HD	Randomization between HDF and high-flux HD
	**Follow-up**	
	• Starts at the randomization (assignment of a strategy, after ≥ 3 months stable high-flux HD treatment)<br>• Ends at death, loss-to-follow-up, or end of the study period whichever came first.	• Starts at the randomization (assignment of a strategy, just before dialysis initiation)<br>• Ends at death, informative censoring due to a competing event (kidney transplantation, recovery of kidney function, withdrawal from dialysis), administrative censoring (end of study or after 5 years) or loss-to-follow-up, whichever came first.
	**Outcomes**	
Primary outcome	All-cause mortality	All-cause mortality
Secondary outcome	• cause-specific mortality<br>• a composite of fatal or nonfatal cardiovascular events<br>• kidney transplantation<br>• recurrent all-cause or infection-related hospitalizations	• cause-specific mortality
Other outcomes	-	• all-cause hospitalizations<br>• cause-specific hospitalizations<br>• proportion of follow-up time spent in hospital<br>• dose–response relationship between convection volume and all outcomes above, in patients receiving HDF
	**Estimands**	
	• Intention-to-treat effect<br>• HR for total follow-up	• Intention-to-treat effect<br>• 5-year survival using a cumulative incidence curve<br>• Restricted mean survival time<br>• HR for the outcome, presented per year for a total of five years
	**Analysis plan**	
	• Intention-to-treat analysis, see Follow-up for censoring.	• Intention-to-treat analysis, see Follow-up for censoring.

HDF, hemodiafiltration; HD, hemodialysis; L, Liters; HR, Hazard Ratio.

Supplemental Table S2: ICD-10 codes for secondary outcomes

**Outcome**	
Non-cardiac cardiovascular cause of mortality/hospitalization	I70.8 (Atherosclerosis of other arteries)<br>I70.2 (Atherosclerosis of arteries of extremities)<br>I71-I74 (Aortic aneurysm and dissection, Other aneurysm and dissection, Other peripheral vascular diseases, Arterial embolism and thrombosis)<br>I60-I64 (Subarachnoid haemorrhage, Intracerebral haemorrhage, Other nontraumatic intracranial haemorrhage, Cerebral infarction, Stroke, not specified as haemorrhage or infarction)<br>I69 (Sequelae of cerebrovascular disease)<br>G45 (Transient cerebral ischaemic attacks and related syndromes)<br>G46 (Vascular syndromes of brain in cerebrovascular diseases)
Cardiac cause of mortality/hospitalization	I20-I25 (Ischaemic heart diseases)<br>I30-I52 (Other forms of heart disease)
Infection-related mortality or hospitalization in- or excluding COVID-19	A00-B99 (Certain infectious and parasitic diseases)<br>J0-J6 (Acute upper respiratory infections)<br>J09-J18 (Influenza and pneumonia)<br>J20-J22 (Other acute lower respiratory infections)<br>*with or without patients with SARS-COV2*
